# Clinicopathologic Characterization of Prostatic Cancer in Dogs

**DOI:** 10.3390/ani14040588

**Published:** 2024-02-10

**Authors:** Demitria M. Vasilatis, Paramita M. Ghosh

**Affiliations:** 1 Department of Urologic Surgery, School of Medicine, University of California Davis, Sacramento, CA 95718, USA; paghosh@ucdavis.edu; 2 Veterans Affairs (VA)—Northern California Healthcare System, Mather, CA 95655, USA; 3 Department of Biochemistry and Molecular Medicine, School of Medicine, University of California Davis, Sacramento, CA 95718, USA

**Keywords:** clinical pathology, cytology, dog, prostatic adenocarcinoma, red blood cell distribution width, red blood cell distribution width to albumin ratio, transitional cell carcinoma, urothelial carcinoma

## Abstract

**Simple Summary:**

Prostatic adenocarcinoma (PRAD) and prostatic transitional cell carcinoma (P-TCC) are the most common subtypes of prostate cancer (PCa) in dogs, and differentiating them often requires an invasive tissue biopsy with histopathology. Routine laboratory data from blood work and minimally invasive tests, such as fine-needle aspiration cytology, have been overlooked as a tool for discerning between these tumors. This is the first study to utilize clinicopathologic and cytologic data to differentiate PRAD and P-TCC in dogs.

**Abstract:**

Clinicopathologic data in dogs with prostate cancer (PCa) may aid in the differentiation between tumor types and subsequent treatment decisions; however, these data are often unreported. Demographic, clinicopathologic, cytologic, histologic and survival data from dogs with primary prostatic adenocarcinoma (PRAD) (*n* = 56) and primary prostatic transitional cell carcinoma (P-TCC) (*n* = 74) were acquired from a tertiary veterinary teaching hospital from 1992 to 2022. Red blood cell distribution width (RDW) to albumin ratio (RAR) was evaluated for diagnostic utility in differentiating between PRAD and P-TCC. Sections from PRAD tumors (*n* = 50) were stained for androgen receptor (AR) expression, and laboratory data were compared between AR positive (AR+) and AR negative (AR−) groups. RDW was increased in PRAD, while albumin was decreased (*p* < 0.05). P-TCC was associated with Melamed-Wolinska bodies (MWB) and necrosis on cytology (*p* < 0.05). RAR had acceptable diagnostic utility in the differentiation of PCa tumors (AUC = 0.7; *p* < 0.05). Survival rates and metastases were equivocal. AR+ and AR− PRAD tumors did not differ in clinicopathologic data or survival (*p* > 0.05). In conclusion, hypoalbuminemia was significantly associated with PRAD and decreased survival, while MWB and necrosis were significantly associated with P-TCC on cytology. These clinicopathologic data may help clinicians differentiate between these tumors ante mortem to guide appropriate treatment and intervention.

## 1. Introduction

Prostate cancer (PCa) in dogs has a poor prognosis, and there is currently no consensus on standard-of-care treatment [1]. Prostatic transitional cell carcinoma (P-TCC) and prostatic adenocarcinomas (PRAD) are the most prevalent PCa in dogs, and the use of nonsteroidal anti-inflammatory drugs (NSAIDs) is often recommended as a first-line treatment; however, this has been largely researched in dogs with tumors of transitional cell (i.e., urothelial) origins [2,3]. The effects of NSAIDs have some conflicting results in human PCa [4,5,6], which are often of glandular origin (i.e., adenocarcinomas) [7]. Though the effect of NSAIDs on dog PRAD specifically is currently unknown, it may be useful to differentiate between P-TCC and PRAD prior to initiating treatment, particularly if pathway-targeting therapeutics are used for pathways enriched in the carcinogenesis of one tumor type and not the other.

One signaling pathway that is often examined prior to the onset of treatment of PCa in humans is the androgen-receptor (AR) signaling pathway. This is variably present in dog PCa; however, one study has shown that up to 40% of TCC in dogs have an AR presence and that revisiting this pathway may be warranted [8,9]. Disruption of this pathway is a mainstay in the initial treatment, management and detection of human PCa and determining whether AR signaling is present in tumors is conducted by measuring serum prostatic specific antigen (PSA) levels [10,11]. In dogs, however, early screening tests for PCa do not exist, and the gold standard for diagnosing and phenotyping PCa is biopsy with histopathology, an invasive procedure that incurs considerable cost and risk [1,12]. Because it is controversial whether dogs have detectable serum PSA, and no species-specific assays currently exist, evaluating other pre-existing routine laboratory data to decipher the AR status of dog tumors could be useful in guiding treatment [13,14].

An additional routine blood work parameter yet to be explored in dog PCa is the red blood cell width (RDW) and red blood cell distribution width to serum albumin ratio (RAR). RDW is a numerical parameter that represents the variation of erythrocyte volume, and an increased RDW alone has been shown as a positive predictor of tumor progression, decreased overall survival and poor treatment outcomes in human PCa [15,16,17,18]. It has also been associated with unfavorable outcomes in the clinical course of other cancers, including breast cancer, hematologic cancers, and osteosarcoma [19,20,21]. RAR is the ratio of RDW to serum albumin, a negative acute phase protein and the most abundant protein in the blood [22]. High RAR ratios in humans have been associated with an all-cause mortality in cancer patients, as well as other non-neoplastic morbidities, including poor prognosis in sepsis and aortic valve replacement [23,24,25]. These data, which are also available on routine blood work in dogs, may help clinicians differentiate and prognosticate PCa in dogs.

The objectives of this study were to determine if discernment between PRAD and P-TCC, as well as PRAD AR+ versus PRAD AR−, could be accomplished with routine laboratory data, minimally invasive fine needle aspiration (i.e., fine needle biopsy) and cytology, and RAR. The results of this study demonstrate that clinicopathologic and cytologic data are useful in differentiating PRAD from P-TCC in dogs.

## 2. Materials and Methods

### 2.1. Dog Selection and Demographic Data

A retrospective electronic medical record search was performed to identify dogs with prostatic cancer. Medical records from the University of California Davis Veterinary Medical Teaching Hospital from January 1992 to May 2022 were investigated. Medical records were searched for all visits by dogs that had a final diagnosis of primary prostate cancer with confirmation by histopathology (i.e., necropsy or biopsy). Cases were excluded if another cancer was present that invaded the prostate secondarily (e.g., primary bladder cancer), if the patient had any unrelated neoplasia in another location, or if the patient had neoplasia of the prostate other than prostatic adenocarcinoma (PRAD) or prostatic transitional cell carcinoma (P-TCC). Data abstracted from the medical records included signalment (i.e., age, castration status, breed), diagnostics performed, co-morbidities, treatment, survival time, and presence of metastases.

### 2.2. Clinicopathologic and Histopathologic Data

Histopathologic and immunohistologic data differentiating between PRAD and P-TCC were also abstracted from the medical records when available. Cases were excluded if the histologic report or immunohistologic results were unable to make a definitive diagnosis. Clinicopathologic data (i.e., complete blood count [CBC], serum biochemistry panel, urinalysis) were also abstracted and recorded from the medical record retrospectively. Hematology analyzers used during the study period included a Baker Systems 9110 Plus (BioChem Immunosystems Inc., Allentown, PA, USA), ADVIA 120 and ADVIA 2120 (Siemens Healthcare Diagnostics Inc., Tarrytown, NY, USA). Biochemistry analyzers used during the study period included a Hitachi 717^C^, Roche Hitachi 917, and Hitachi Cobas c501 (Roche Diagnostics Corporation, Indianapolis, IN, USA). Results were calibrated when instruments were upgraded to maintain consistency in results between analyzers. Semiquantitative urinalysis dipstick data were converted to their equivalent quantitative values per dipstick manufacturer instructions (e.g., “+1 or +2” protein was recorded as 75 mg/dL). Values recorded as “trace” were considered negative. Mean values were recorded when ranges were provided for components of the urine sediment examination (e.g., white blood cell [WBC] sediment counts recorded as “5–9 WBC/field” were noted as 7 WBC/field). Cytologic data and specimens from May 2012 (which was the earliest year slides were available to re-examine) to May 2022 were reviewed and recorded by a board-certified veterinary clinical pathologist (D.M.V.), and observations of interest were imaged. These observations of interest were recorded as either present or absent, and they included Melamed–Wolinska bodies, necrosis, inflammation, mineralization, vacuolation of neoplastic cells, mitotic figures, keratinization of neoplastic cells and presence of hemosiderin. Receiver operating characteristic (ROC) curves were performed to evaluate the diagnostic utility of the red blood cell distribution width to albumin ratio (RAR), RDW and ALB as diagnostic tests to differentiate PRAD from P–TCC.

### 2.3. Immunohistochemistry Staining for Androgen Receptors and Laboratory Data Evaluation

FFPE sections from PRAD tumors were requested from January 1992 to May 2022 and sectioned. Immunohistochemistry was conducted as previously described [26], and AR (dilution 1:150; N-20, Santa Cruz Biotechnology, Inc., Dallas, TX, USA) expression and location (cytosolic vs. nuclear) was recorded. Positivity for expression was defined as staining in ≥ 10% of neoplastic cells [27]. Tumors were considered AR positive (AR+) if the nuclei or cytoplasm were positive for expression. Normal prostate tissues from intact dogs were used as a positive control. Baseline laboratory data (i.e., CBC, serum biochemistry, and urinalysis) from dogs with AR+ PRAD tumors were compared to dogs with AR negative (AR−) PRAD tumors to determine if any analyte was suitable as a biomarker for androgen receptor signaling ante mortem. Additionally, ROC curves were performed to evaluate the diagnostic utility of RAR as a test to differentiate AR+ from AR− PRAD tumors.

### 2.4. Statistical Analysis

Data was downloaded into a Microsoft Excel spreadsheet and analyzed in GraphPad Prism version 10.1.0. Descriptive statistics for categorical variables were reported as frequency or frequency with percentage, while continuous variables were reported as median with range (i.e., minimum-maximum). The association between categorical variables was assessed by chi-squared test or Fisher’s exact test, pending sample size of each observation (*n* < 5, Fisher’s exact test; *n* ≥ 5, chi-squared test). Normality of laboratory data was determined by a Shapiro–Wilk test. Non-parametric data were compared with a Mann–Whitney test for data separated into two groups, or a Kruskal–Wallis test with a post hoc Dunn’s multiple comparison test when data were separated into three groups. Red blood cell distribution width to albumin ratio (RAR) diagnostic utility was determined by a receiver–operator curve (ROC), and test cut-off value was determined using the value closest to (0,1) criterion. The Youden index was also calculated to confirm diagnostic utility. Patient survival times were measured from onset of clinical signs (time = 0) until death unless otherwise noted. Kaplan–Meier analyses were compared by a Mantel–Cox log-rank test. A multivariate Cox proportional hazards regression model was used to compare RDW, ALB and RAR with survival data in PRAD and P-TCC with survival >1 day. A *p*-value of <0.05 was considered significant.

## 3. Results

### 3.1. Medical Record Inclusions and Exclusions

There were 150 records found within the study period that had a final diagnosis of primary prostatic cancer confirmed by histology (necropsy or biopsy). Eleven records were excluded for neoplasia (amelanotic melanoma [*n* = 1], anal sac gland adenocarcinoma [*n* = 3], anaplastic carcinoma [*n* = 5], colonic carcinoma [*n* = 1], and gastric carcinoma [*n* = 1]) other than P-TCC or PRAD in the prostate. Eight records were excluded because another neoplastic process was present concurrently and was the primary cause for presentation (anal sac gland adenocarcinoma [*n* = 1], nasal B-cell lymphoma [*n* = 1], chemodectoma [*n* = 1], oligodendroglioma [*n* = 1], melanoma [*n* = 1], rhabdomyosarcoma [*n* = 1], thyroid carcinoma [*n* = 1] and histiocytic sarcoma [*n* = 1]). One record was excluded because neoplasia was not found in the prostate and a bladder mass was mistakenly believed to be a prostatic mass ante-mortem. Ultimately, 130 records of patients with primary prostate tumors that were confirmed by either biopsy (*n* = 21) or necropsy (*n* = 109) were included in this study. Seventy-one specimens (9 biopsy, 62 necropsy) had immunohistochemistry performed to reaffirm the diagnosis (i.e., prostatic-specific acid phosphatase [PSAP], cytokeratin 7 [CK7], cytokeratin 20 [CK20], uroplakin 3 [UPKIII]). A diagnosis of PRAD was made if glandular epithelial cells were the neoplastic cells of origin or neoplastic cells were positive for expression of PSAP on IHC. A diagnosis of P-TCC was made if prostatic ductal epithelium or prostatic urethral epithelium were the neoplastic cells of origin or neoplastic cells were positive for expression of UPKIII, CK7 or CK20. If IHC staining was equivocal, a definitive diagnosis was made based on the pathologist’s overall impression. Of the 130 records, 74/130 (56.9%) received a final histopathologic diagnosis of P-TCC, while 56/130 (43.1%) were deemed PRAD. Most dogs affected by PRAD or P-TCC were castrated, large mixed-breeds, and both groups had a median age of 10.1 years. German shepherds and American Pit Bull terriers were the most prevalent purebreds in PRAD, while Labrador retrievers and Scottish terriers were the most prevalent purebreds in P-TCC. Additional age, sex, and breed distributions for each tumor type were recorded (Table 1 and Table 2).

### 3.2. Clinicopathologic Data Results

Of the 56 PRAD dogs, 32 had a CBC performed, 29 had a serum biochemistry panel performed, 25 had a urinalysis performed, and 24 had fine-needle aspirate cytology performed at initial presentation. Three cytologies were excluded because they were from metastatic sites and not the primary tumor, resulting in a final number of 21 cytologies for PRAD. Of the 74 P-TCC dogs, 34 had a CBC performed, 31 had a serum biochemistry panel performed, 30 had a urinalysis performed and 25 had fine-needle aspirate cytology performed at initial presentation.

#### 3.2.1. Complete Blood Count (CBC), Serum Biochemistry and Urinalysis

Regarding CBC, RDW% was significantly increased in PRAD compared to P-TCC but within reference range (*p* = 0.02) (Table 3), while on-serum biochemistry albumin was mildly decreased below the reference interval in PRAD compared to P-TCC (*p* = 0.03) (Table 4).

The RDW to albumin ratio (RAR) was also evaluated, with PRAD having a significantly higher RAR value compared to P-TCC (*p* = 0.01). An ROC curve of RAR data to differentiate PRAD from P-TCC resulted in an AUC of 0.70 (95% CI: 0.56–0.85); *p* = 0.01); a cut-off score of >4.00 (sensitivity [Sn] = 60.0% [95% CI: 40.7–76.7%]; specificity [Sp] = 66.7% [95% CI: 48.8–80.8%]; likelihood ratio: 1.8) using the closest to (0,1) criterion; and had a Youden index of 0.27. The highest Youden index (0.35) in the RAR data resulted in a cut-off score of >4.850 (Sn = 48.0% [95% CI: 30.0–66.5%]; Sp = 86.7% [95% CI: 70.3–94.7%]; likelihood ratio: 3.6). In addition, ROC curves of ALB and RDW were also performed to examine their diagnostic utility individually in differentiating PRAD from P-TCC. An ROC curve of ALB data resulted in an AUC of 0.66 (95% CI: 0.53–0.80; *p* = 0.03), a cut-off score of <3.4 g/dL (Sn = 65.6% [95% CI: 47.4–80.1%]; Sp = 54.8% [95% CI: 37.8–70.8%]; likelihood ratio: 1.5); and a Youden index of 0.20. An ROC curve of RDW resulted in an AUC of 0.67 (95% CI: 0.54–0.81; *p* = 0.02), a cut-off score of >13.25% (Sn = 59.4% [95% CI: 42.3–74.5%]; Sp = 61.8% [95% CI: 45.0–76.1%]; likelihood ratio: 1.6); and a Youden index of 0.21 (Figure S1).

There were no significant differences in urinalysis interpretation or quantitative data between the groups, apart from isosthenuria being associated with P-TCC (*p* = 0.01) (Table 5 and Table 6).

#### 3.2.2. Fine-Needle Aspirate Cytology of PRAD and P-TCC

**Figure 1:**
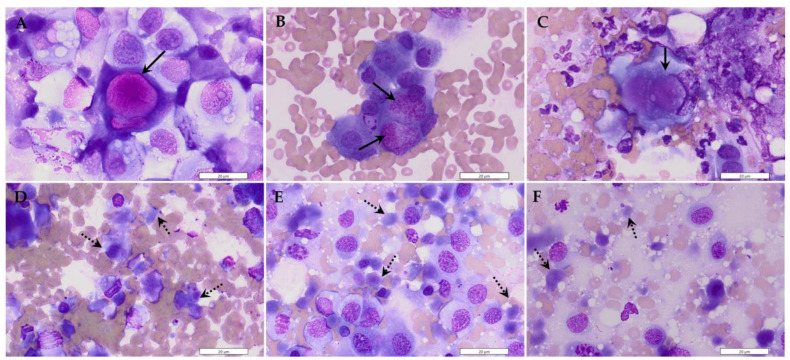
Cytologic features of P-TCC. (**A**–**C**) P-TCC was significantly associated with Melamed–Wolinska bodies, large eosinophilic globular inclusions in neoplastic cells (solid arrows). (**D**–**F**) P-TCC was also significantly associated with necrosis, which are grey–blue amorphous cellular remnants with deteriorated nuclei (dashed arrows). Panels (**A**–**F**), 100× oil objective.

Cytology specimens from PRAD and P-TCC tumors were evaluated for features typically associated with urogenital tumors (e.g., necrosis, vacuolation, etc.) and enumerated for each specimen. Melamed–Wolinska bodies (MWB) (*p* = 0.02) and necrosis (*p* = 0.03) were significantly associated with P-TCC (Figure 1), while all other features were not significantly associated with either neoplasia (Table 7).

### 3.3. Androgen Receptor Immunohistochemistry and Clinicopathologic Data Results

**Figure 2:**
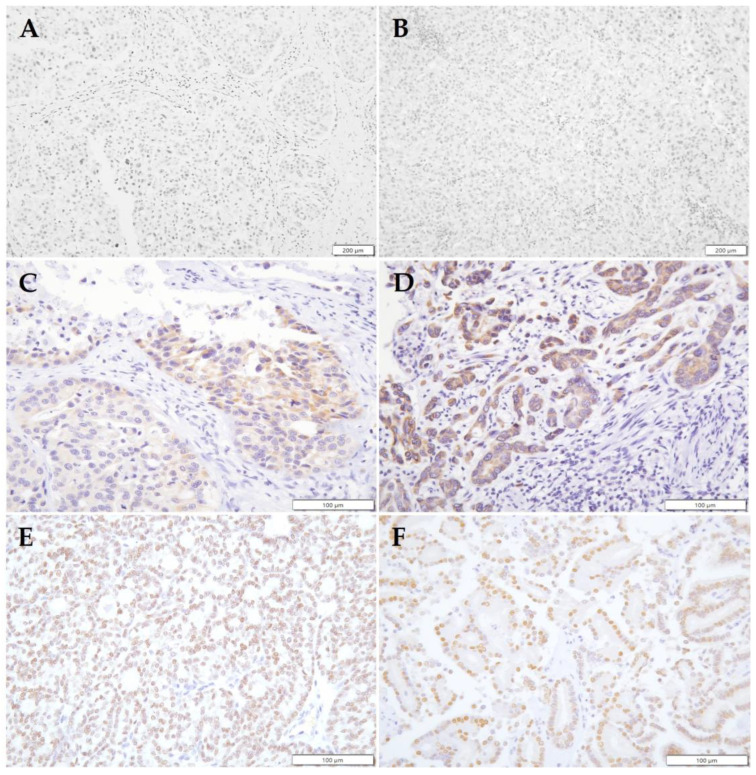
AR expression in PRAD tumors. (**A**,**B**) The majority of PRAD is negative for AR, with few tumors displaying AR cytoplasmic staining (**C**,**D**). Fewer cases of PRAD tumors have AR positive nuclei (**E**,**F**), suggesting dog PRAD tumors do not often utilize canonical AR signaling. Panels (**A**,**B**), 20× objective; panels (**C**–**F**), 40× objective.

Sections from 50 PRAD dogs were available for androgen receptor (AR) immunohistochemical staining. Six dogs did not have enough tissue left from their archived samples for sectioning. Seven dogs (14%) had tumors with AR positive nuclei, 14 dogs (28%) had tumors with AR positive cytoplasm, and 29 dogs (58%) were negative for AR staining in either location (Figure 2).

Of the 50 dogs with FFPE blocks, 30 had CBC performed, 28 had serum biochemistry performed, and 24 had urinalysis performed. The clinicopathologic data were not significant between AR+ (positive nuclear or cytoplasmic staining) and AR− (negative staining) tumor sections (*p* > 0.05), apart from higher-circulating monocytes (*p* = 0.02) and aspartate transaminase (AST) (*p* = 0.01) values in dogs with AR− tumors (Tables S1–S4), but these were not above the reference interval. The ROC curve of RAR data to differentiate between AR+ from AR− PRAD tumors was not significant (*p* > 0.05) (Figure S2).

### 3.4. Survival Times in Prostate Cancer

Survival times were not attainable for 15 records (8 PRAD, 7 P-TCC), with four necropsy specimens having no mention of a start date of symptoms in the medical records and 11 biopsy specimens having no mention of death or euthanasia because the patient was lost to follow-up. Nearly 40%of patients were euthanized less than 24 h after diagnosis for both groups (Table S5). Notably, 48 PRAD and 67 P-TCC dogs were included in survival analysis, with a median survival time (MST) of 3 days for PRAD and 17 days for P-TCC. When animals that survived less than 24 h were excluded, the MST of PRAD increased to 35 days and the MST of P-TCC increased to 45 days, respectively. There was no significant difference in survival times between the groups (*p* > 0.05) (Figure S3). Moreover, there were no significant differences in survival times between dogs with AR+ and AR− PRAD tumors, and they featured an overall MST of 2 and 3.5 days, respectively (*p* > 0.05) (Figure S4). This increased when animals that survived less than 24 h were excluded to 18.5 days and 35 days for AR+ and AR−, respectively. Additional information regarding survival times for PRAD and P-TCC is provided in Supplementary Table S5. Lastly, Cox regression multivariate modeling revealed hypoalbuminemia (hazard ratio [HR] 11.79 [95% CI: 1.99–102.9]; *p* = 0.01) as a risk factor for shorter survival in PRAD, but not increased RDW or RAR. These variables did not affect survival in P-TCC (*p* > 0.05) (Table S6).

## 4. Discussion

In this study, we found that hypoalbuminemia in serum biochemistry was significantly associated with PRAD, while MWB and necrosis were significantly associated with P-TCC on cytology. In addition to this, we found RDW was significantly increased in PRAD when compared to P-TCC, and that RAR could be used with acceptable diagnostic utility to differentiate PRAD from P-TCC when ratio values were >4.00. These findings have important implications for the use of routine, minimally invasive diagnostic tests to distinguish different subtypes of PCa in dogs in order to guide appropriate treatment and intervention.

Increased RDW, which represents anisocytosis in the erythrocyte population, is normally present in regenerative anemias due to the presence of reticulocytes, or in iron-deficiency anemias due to the presence of microcytes. It has also been observed in inflammatory states in humans where erythrocyte fragmentation, altered erythrocyte morphology, impaired erythrocyte maturation or extended erythrocyte lifespan may be affected by pro-inflammatory cytokines, leading to increased heterogeneity in erythrocyte volume [18,24,28]. Though still under investigation, this has been supported by other studies showing a significant relationship between RDW and C-reactive protein and leukocyte counts in humans as well as dogs [29,30,31,32,33]. Because local and/or systemic inflammation is commonplace with PCa and other cancers, we suspect this is why the median RDW value was at the higher end of the reference range in dog PCa [34,35]. RDW values were significantly higher in PRAD (*p* < 0.05) than P-TCC, but median values were within the reference interval, thereby limiting the utility of this hematologic parameter in differentiating PRAD from P-TCC.

Additionally, RAR is a novel simple biomarker of inflammation that evaluates RDW and albumin, with high RAR values associated with increased RDW and decreased albumin [25]. This parameter is largely underutilized in veterinary medicine, and our study is one of the first to investigate its utility in dogs. Ultimately, the diagnostic benefit of RAR to discern PRAD from P-TCC is acceptable when the value is >4.00, but a value of >4.850 may be of better diagnostic use with a higher Youden index and specificity, though with a lower sensitivity. Moreover, RAR had a slightly better AUC and Youden index than ALB or RDW alone and may be more useful for differentiation of dog PCa. Taken together, these data imply that PRAD has a greater effect on hematologic parameters and inflammation than P-TCC, which is supported by the significant hypoalbuminemia in PRAD. However, this assumption could benefit from prospective studies with additional confirmatory diagnostics, such as serum testing for C-reactive protein, interleukin 6 (IL-6) and tumor necrosis factor alpha (TNF-α) levels. Moreover, despite its use for prognostication in survival times for cancer in humans, RAR did not appear to significantly predict survival outcomes in PRAD or P-TCC in this study, though hypoalbuminemia significantly predicted survival times in PRAD, which has also been demonstrated in human studies [36,37,38]. Therefore, further studies investigating the utility of RAR to predict survival outcomes are warranted.

Hypoalbuminemia in dogs may be found through increased losses (e.g., protein-losing enteropathy, protein-losing nephropathy, hemorrhage), decreased production (i.e., liver failure, inflammation [negative acute phase protein]), increased sequestration or third-spacing (i.e., protein-rich body cavity effusions), and with a negative energy balance (i.e., catabolism) [39,40]. None of the medical records in this study or laboratory data indicated that protein-losing enteropathy, protein-losing nephropathy, body cavity effusions or liver failure were present in either the PRAD or P-TCC groups. Furthermore, there was no difference in hematuria or proteinuria between the groups. As such, hypoalbuminemia in PRAD suggests that it incites more inflammation than P-TCC, although CBC leukocyte values and cytology findings between the groups do not entirely support the latter. Future studies evaluating inflammatory cytokine levels between these groups may be necessary to support this suspicion.

Cytologic evaluation of prostatic tissue specimens via fine needle aspiration has a strong agreement with histopathologic diagnosis and is a less invasive diagnostic modality in diagnosing prostatic neoplasia [41,42]. In this study, P-TCC was significantly associated with the presence of MWB and necrosis on cytology, but other cytologic features (e.g., inflammation, vacuolation) did not significantly differ between the groups. MWB are intracytoplasmic inclusions found within degenerating benign and malignant urothelial cells, and they appear as large, pink-red, globular material on cytology without the need for special stains [43]. These structures have been predominantly associated with urothelial carcinoma in humans and dogs but are not pathognomonic [44,45]. In regard to histology, MWB usually appear as large clear vacuoles or vacuoles with faint pink stippling, but they can be highlighted with Period-acid Schiff (PAS) special stain [45,46]. This finding in our study is supported by other accounts of MWB associated with urothelial carcinomas in dogs [47,48]. Necrosis is a non-specific and common finding in neoplasia, but it was more prevalent in P-TCC than PRAD in our study. This may be owing to the confined location of P-TCC arising from the centralized urethra and collecting ducts of the prostate, which may have caused tissue compression and subsequent ischemic necrosis, but additional histopathologic-based studies are needed to further evaluate this observation.

Limitations to our study include those associated with retrospective data collection, including missing data in charts, loss of follow up, inherent selection bias, and lack of standardization of treatment or diagnostic investigation of patients [49]. Survival times in this study, for instance, may have been shortened compared to other studies in the literature, as the requirement for histopathologic diagnosis may have biased the case selection towards dogs that were euthanized and necropsied at a tertiary referral institution. Moreover, a large portion of patients were euthanized <24 h. Additionally, because the data was acquired from a tertiary referral hospital and may not represent the breadth of clinicopathologic parameters that patients with P-TCC and PRAD may have. In addition, retrospective laboratory data was not able to be confirmed by follow-up testing or pathologist review (e.g., confirm proteinuria, confirm RBCs per HPF on sediment examination). Furthermore, three different hematology and chemistry analyzers were utilized over the 30-year time span of the laboratory data, which may have introduced variability into the values reported. Moreover, the hypoalbuminemic values reported in this study may be considered within reference range at other institutions depending on their laboratory’s instrument validation protocols and reference intervals. A limitation of the study was the small sample size used when comparing the laboratory data of AR+ to AR− PRAD patients, as well as the regression analysis of the effect of RAR, RDW and ALB on survival times, which may result in type II statistical errors. Lastly, P-TCC was not explored for AR status and association with blood work parameters, and so remains a viable opportunity for further exploration.

## 5. Conclusions

In summary, hypoalbuminemia was significantly associated with PRAD and decreased survival, while MWB and necrosis were significantly associated with P-TCC on cytology. RAR is modestly helpful in differentiating PRAD from P-TCC. These clinicopathologic data may help clinicians differentiate between these tumors ante mortem to guide appropriate treatment and intervention, especially if novel pathway inhibitors are being explored. Future studies evaluating the AR status of P-TCC and its clinicopathologic associations, as well as the ability of RAR to prognosticate outcomes for other tumor types in dogs, may be of interest to investigators and veterinary oncologists to help promote patient welfare.

## Figures and Tables

**Table 1:** Age and neuter status of dogs with PRAD and P-TCC.

		PRAD (*n* = 56)	P-TCC (*n* = 74)
Age	Young Adult (0–5 years)	3 (5%)	2 (3%)
	Mature Adult (6–10 years)	26 (46%)	42 (57%)
	Geriatric (11–15 years)	21 (38%)	22 (30%)
	Unknown	6 (11%)	8 (11%)
Neuter Status	Intact	15 (27%)	12 (16%)
	Castrated	41 (73%)	62 (84%)

**Table 2:** Top 5 breeds of dogs with PRAD and P-TCC.

Tumor Type	Breeds	N (%)
PRAD (*n* = 56)	Large Mixed Breed (>50 lbs)	8 (14%)
	German Shepherd	5 (9%)
	American Pit Bull Terrier	5 (9%)
	Labrador Retriever	3 (5%)
	Medium Mixed Breed (20–49 lbs)	3 (5%)
P-TCC (*n* = 74)	Large Mixed Breed (>50 lbs)	13 (18%)
	Labrador Retriever	7 (9%)
	Scottish Terrier	6 (8%)
	German Shepherd	4 (5%)
	Golden Retriever	4 (5%)

**Table 3:** Complete Blood Cell Count Findings for PRAD and P-TCC.

Variable	PRAD (*n* = 32)	P-TCC (*n* = 34)	Ref. Int.	*p*-Value
RBC (×10^6^/µL)	6.06 (2.97–7.57)	6.54 (4.22–7.8)	5.6–8	n.s.
HGB (g/dL)	14.2 (6.9–19.5)	15.3 (10.1–17.8)	14–19	n.s.
HCT (%)	41 (21.4–52.5)	43 (29.2–52.5)	40–55	n.s.
MCV (fL)	68.9 (59.9–75.6)	67.3 (58.3–73.9)	65–75	n.s.
MCH (pg)	23.8 (20.4–28.7)	23.4 (20.6–25.5)	22–26	n.s.
MCHC (g/dL)	34.5 (32.2–38.3)	34.6 (33–37.5)	33–36	n.s.
RDW (%) *	13.95 (11.3–19.7)	12.9 (11.5–15.7)	11–14	0.02
RETIC (/µL)	0 (0–155,400)	0 (0–129,800)	7000–65,000	n.s.
nRBC (/100 WBC)	0 (0–3)	0 (0–5)	RARE	n.s.
WBC (/µL)	13,717 (6610–34,772)	12,190 (2780–53,500)	6000–13,000	n.s.
Neutrophils (/µL)	11,228 (4158–31,295)	9935 (1918–48,632)	3000–10,500	n.s.
Immature Neutrophils (/µL)	0 (0–2576)	0 (0–1725)	RARE	n.s.
Lymphocytes (/µL)	1109 (409–7523)	1394 (417–2954)	1000–40,000	n.s.
Monocytes (/µL)	892 (258–3492)	864 (241–3549)	150–1200	n.s.
Eosinophils (/µL)	218 (0–959)	129 (0–1341)	0–1500	n.s.
Basophils (/µL)	0 (0–898)	0 (0–107)	0–50	n.s.
PLT (×10^3^/µL)	277 (19–717)	295 (54–876)	150–400	n.s.
MPV (fL)	9.9 (7–17.6)	10 (7.6–16.7)	7.0–13.0	n.s.
TP (g/dL)	7.1 (6.1–8.9)	7.2 (5.5–9.1)	6.0–8.0	n.s.

Data are median (min–max). A Mann–Whitney test was used to compare differences in CBC data; ref. int., reference interval; n.s., no significant difference; * CBC interpretations that are significantly associated with PRAD.

**Table 4:** Serum Biochemistry Findings for PRAD and P-TCC.

Variable	PRAD (*n* = 29)	P-TCC (*n* = 31)	Ref. Int.	*p*-Value
Anion Gap (mmol/L)	21 (13–36)	21 (16–39) ^†^	12–20	n.s.
Sodium (mmol/L)	146 (138–153)	147 (114–166) ^†^	143–151	n.s.
Potassium (mmol/L)	4.3 (2.8–6.7)	4.4 (3.6–7.4) ^†^	3.6–4.8	n.s.
Chloride (mmol/L)	111 (89–126)	110 (72–129) ^†^	108–116	n.s.
Total CO_2_ (mmol/L)	19 (11–29)	20 (10–26) ^†^	20–29	n.s.
Phosphorus (mg/dL)	4.7 (2.4–14.0)	4.8 (2.7–44)	2.6–5.2	n.s.
Calcium (mg/dL)	10.2 (8.3–12.4)	10.2 (2.6–15.2)	9.6–11.2	n.s.
BUN (mg/dL)	19 (6–152)	19 (0.7–186)	11–33	n.s.
Creatinine (mg/dL)	1.1 (0.5–11.1)	0.9 (0.5–44.0)	0.8–1.5	n.s.
Glucose (mg/dL)	103 (1.8–170) ^‡^	107 (77–158)	86–118	n.s.
Total protein (g/dL)	6.2 (4.9–7.6)	6.3 (4.9–8.0)	5.4–6.9	n.s.
Albumin (g/dL) *	3.2 (1.7–4.0)	3.5 (2.0–4.6)	3.4–4.3	0.03
Globulins (g/dL)	3.1 (2.1–5.2)	2.9 (2.0–4.1)	1.7–3.1	n.s.
ALT (U/L)	42 (19–362) ^‡^	40 (9–183)	21–72	n.s.
AST (U/L)	31 (15–288) ^‡^	26 (14–113)	20–49	n.s.
CK (U/L)	199 (76–873) ^‡^	115 (72–591) ^†^	55–257	n.s.
ALP (U/L)	103 (16–1395) ^‡^	89 (15–2531)	14–91	n.s.
GGT (U/L)	4 (0–20) ^‡^	2 (0–20)	0–5	n.s.
Cholesterol (mg/dL)	246 (117–398) ^‡^	245 (146–351)	139–353	n.s.
Total Bilirubin (mg/dL)	0.1 (0.0–22.1) ^‡^	0.1 (0.0–126.0)	0.0–0.2	n.s.
Magnesium (mg/dL)	2.1 (1.7–3.2) ^‡^	2.1 (1.7–2.5) ^†^	1.9–2.5	n.s.

Data are median (min–max). A Mann–Whitney test was used to compare differences in biochemistry data between tumor types; ref. int., reference interval; n.s., no significant difference; * Biochemistry interpretations that are significantly associated with PRAD; ^†^ Fewer *n* were evaluated for these analytes in the P-TCC group: Anion Gap, Sodium, Potassium, Chloride, Total CO_2_, *n* = 30; Creatinine Kinase, *n* = 19; Magnesium, *n* = 20; ^‡^ Fewer *n* were evaluated for these analytes in the PRAD group: Glucose, ALT, AST, ALP, CHOL, Total Bilirubin, *n* = 27; CK, *n* = 16; GGT, *n* = 24; Magnesium, *n* = 11.

**Table 5:** Urinalysis quantitative results for PRAD and P-TCC.

Variable	PRAD (*n* = 25)	P-TCC (*n* = 30)	Ref. Int.	*p*-Value
pH	6.5 (5.0–8.5)	6.5 (5.0–9.0)	5.0–9.0	n.s.
USG	1.027 (1.010–1.049)	1.021 (1.002–1.070)	1.001–1.060	n.s.
Protein (mg/dL)	75 (0–500)	75 (0–500)	NEG	n.s.
Bilirubin (mg/dL)	1 (0–6)	1 (0–3)	NEG	n.s.
Hemoprotein (ery./µL)	250 (0–350)	250 (10–250)	NEG	n.s.
WBC (/HPF)	7 (0–500)	8 (0–100)	0–3	n.s.
RBC (/HPF)	45 (0–250)	39 (1–100)	0–2	n.s.

Data are median (min–max). A Mann–Whitney test was used to compare differences in urinalysis quantitative data between tumor types; ery., erythrocytes; ref. int., reference interval; NEG, negative; n.s., no significant difference. No animals had glucosuria or ketonuria.

**Table 6:** Frequency of urinalysis interpretation and association with PRAD and P-TCC.

Interpretation	PRAD (*n* = 25)	P-TCC (*n* = 30)	Ref. Int.	*p*-Value
Alkaluria	5 (20%)	5 (17%)	pH > 7.5	n.s.
Aciduria	2 (8%)	4 (13%)	pH < 6.0	n.s.
Adequate USG	23 (92%)	21 (70%)	1.014 < USG < 1.030	n.s.
Isosthenuria *	0 (0%)	7 (23%)	USG 1.007–1.013	0.01
Hyposthenuria	2 (8%)	2 (7%)	USG < 1.007	n.s.
Proteinuria	15 (60%)	22 (73%)	>75 mg/dL	n.s.
Bilirubinuria	15 (60%)	15 (50%)	>0 mg/dL	n.s.
Hematuria	22 (88%)	28 (93%)	>2 RBC/HPF	n.s.
Pyuria	14 (56%)	20 (67%)	>3 WBC/HPF	n.s.
Crystalluria	10 (40%)	5 (17%)	NONE	n.s.
Casts	8 (32%)	3 (10%)	NONE	n.s.
Epithelial cells	24 (96%)	28 (93%)	NONE	n.s.
Bacteriuria	5 (20%)	4 (13%)	NONE	n.s.

Data includes the number of animals (percentage of animals) with that urinalysis interpretation; the chi-squared test performed when all observations *n* ≥ 5; Fisher’s exact test performed when any observation *n* < 5; n.s., no significant difference; ref. int., reference interval. *Urinalysis interpretations that are significantly associated with P-TCC.

**Table 7:** Cytologic features associated with PRAD and P-TCC.

Cytologic Feature	PRAD (*n* = 18)	P-TCC (*n* = 22)	*p*-Value
Melamed-Wolinska bodies *	5 (28%)	14 (63%)	0.02
Necrosis *	3 (17%)	11 (50%)	0.03
Inflammation	3 (17%)	6 (27%)	n.s.
Mineralization	1 (6%)	2 (9%)	n.s.
Vacuolation	7 (39%)	6 (27%)	n.s.
Mitotic Figures	2 (11%)	3 (14%)	n.s.
Keratinization	3 (17%)	3 (14%)	n.s.
Hemosiderin	3 (17%)	1 (5%)	n.s.

Data includes the number of animals (percentage of animals) with that feature on their cytology specimen; chi-squared test performed when all observations *n* ≥ 5; Fisher’s exact test performed when any observation *n* < 5; n.s., no significant difference; * Cytological features that are significantly associated with P-TCC.

## Data Availability

The data presented in this study are available on request from the corresponding author.

## Supplementary Materials

The following supporting information can be downloaded at: https://www.mdpi.com/article/10.3390/ani14040588/s1, Figure S1: RAR is of acceptable diagnostic utility in distinguishing PRAD from P-TCC; Table S1: Complete blood cell count findings for AR+ and AR− PRAD tumors; Table S2: Serum biochemistry findings for AR+ and AR− PRAD tumors; Table S3: Quantitative urinalysis findings for AR+ and AR− PRAD tumors; Table S4: Urinalysis interpretations and associations for AR+ and AR− PRAD tumors; Figure S2: RAR is not significant in distinguishing AR+ from AR− PRAD; Figure S3: Survival times do not significantly differ between PRAD and P-TCC; Figure S4: Survival times do not significantly differ between AR+ and AR− PRAD tumors; Table S5: Ranges of survival times in PRAD and P-TCC dogs; Table S6: Multivariate survival analysis of 13 PRAD and 20 P-TCC dogs surviving >1 day.
