# Supplementary material for: Clinicopathologic Characterization of Prostatic Cancer in Dogs
Source: Animals (Basel). 2024 Feb 10;14(4):588. doi: 10.3390/ani14040588 (PMC10886256; doi:10.3390/ani14040588)
Supplement: Supplementary file 1 [file animals-14-00588-s001.zip › animals-2829119-supplementary.pdf]

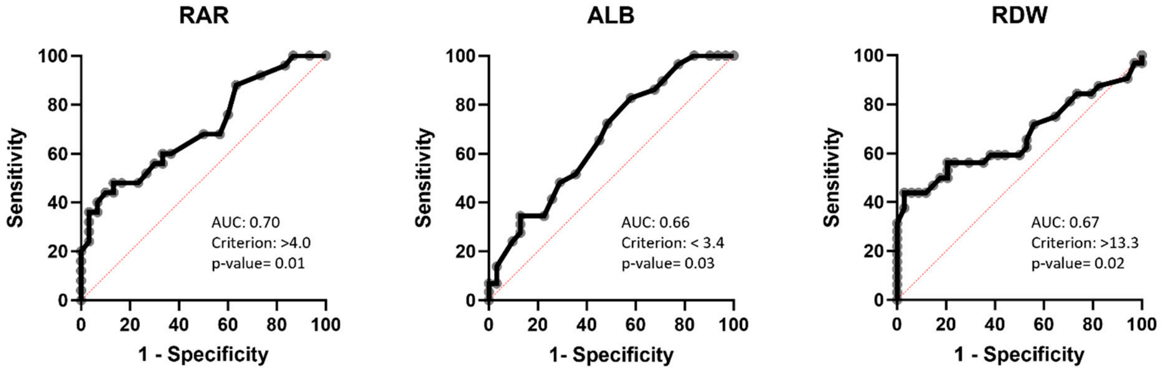

Figure S1: RAR is of acceptable diagnostic utility in distinguishing PRAD from P-TCC. Using an RAR cut-off value of >4.0 distinguishes PRAD from P-TCC using the (0,1) criterion, while a cut-off value of >4.850 is suggested when using the highest Youden index (0.35) for the ROC curve.

Variable	AR+ (n=9)	AR- (n=21)	Ref. Int.	p-value
RBC (×10^6/μL)	6.0 (3.8-7.4)	6.1 (3.0-7.6)	5.6–8.0	n.s.
HGB (g/dL)	14.3 (9.6-18.0)	14.2 (6.9-19.5)	14–19	n.s.
HCT (%)	41.8 (25.4-50.7)	41.2 (21.4-52.5)	40–55	n.s.
MCV (fL)	67.7 (61.7-75.6)	69.0 (59.9-75.0)	65–75	n.s.
MCH (pg)	23.7 (21.5-26.5)	23.8 (20.4-28.7)	22–26	n.s.
MCHC (g/dL)	34.7 (32.4-38.2)	34.6 (32.2-38.3)	33–36	n.s.
RDW (%)	12.9 (11.8-19.7)	14.2 (11.30-18.7)	11–14	n.s.
RETIC (/μL)	0 (0-155,400)	0 (0-154,700)	7000–65,000	n.s.
nRBC (/100 WBC)	0 (0-0)	0 (0-3)	RARE	n.s.
WBC (/μL)	10,900 (6610-22,230)	16,500 (7970-34,772)	6000–13,000	n.s.
Neutrophils (/μL)	9156 (4158-18,509)	12,098 (5252-31,295)	3000–10,500	n.s.
Immature Neutrophils (/μL)	0 (0-446)	0 (0-2576)	RARE	n.s.
Lymphocytes (/μL)	1109 (503-2230)	975 (409-7523)	1000–40,000	n.s.
Monocytes (/μL) *	637 (258-1247)	1082 (409-3492)	150–1200	0.02
Eosinophils (/μL)	218 (101-483)	231 (0-959)	0–1500	n.s.
Basophils (/μL)	1 (0-101)	5 (0-898)	0–50	n.s.

PLT ($\times 10^3/\mu L$)	266 (163-546)	272 (19-717)	150–400	n.s.
MPV (fL)	10 (8.2-13.0)	9.9 (7.0-17.6)	7.0–13.0	n.s.
TP (g/dL)	6.7 (6.2-8.9)	7.1 (6.1-8.3)	6.0–8.0	n.s.

Table S1: Complete blood cell count findings for AR+ and AR- PRAD tumors. There were no significant differences in CBC parameters between the groups apart from slightly higher circulating monocytes in AR- group (p=0.02).

Variable	AR+ (n=8)	AR- (n=20)	Ref. Int.	p-value
Anion Gap (mmol/L)	21 (13-36)	20 (13-34)	12–20	n.s.
Sodium (mmol/L)	146 (142-152)	146 (138-153)	143–151	n.s.
Potassium (mmol/L)	4.2 (3.8-6.3)	4.4 (2.8-6.7)	3.6–4.8	n.s.
Chloride (mmol/L)	111 (103-115)	112 (89-126)	108–116	n.s.
Total CO_2 (mmol/L)	19 (11-22)	19 (11-29)	20–29	n.s.
Phosphorus (mg/dL)	6.3 (5.7-12.8)	4.5 (2.4-14.0)	2.6–5.2	n.s.
Calcium (mg/dL)	9.9 (8.3-12.4)	10.3 (8.5-11.6)	9.6–11.2	n.s.
BUN (mg/dL)	9 (6-152)	22 (6-93)	11–33	n.s.
Creatinine (mg/dL)	0.9 (0.5-11.1)	1.1 (0.5-4.6)	0.8–1.5	n.s.
Glucose (mg/dL)	104.5 (86-131)	103 (18-170)	86–118	n.s.
Total protein (g/dL)	5.95 (4.9-7.6)	6.3 (5.5-7.5)	5.4–6.9	n.s.
Albumin (g/dL)	3.1 (1.8-3.9)	3.3 (1.7-4.0)	3.4–4.3	n.s.
Globulins (g/dL)	3.4 (2.1-4.4)	3.0 (2.2-5.2)	1.7–3.1	n.s.
ALT (U/L)	41 (19-62)	43 (21-362)	21–72	n.s.
AST (U/L) *	24 (15-33)	38 (19-288)	20–49	0.01
CK (U/L)	137 (76-152)	293 (119-873)	55–257	n.s.
ALP (U/L)	83 (16-365)	112 (17-1395)	14–91	n.s.
GGT (U/L)	4 (0-9)	5 (0-20)	0–5	n.s.
Cholesterol (mg/dL)	254 (163-336)	246 (117-398)	139–353	n.s.
Total Bilirubin (mg/dL)	0.2 (0.0-0.3)	0.1 (0.0-22.1)	0.0–0.2	n.s.
Magnesium (mg/dL)	2.7 (2.1-3.2)	2.1 (1.7-2.3)	1.9–2.5	n.s.

Table S2: Serum biochemistry findings for AR+ and AR- PRAD tumors. There were no significant differences in biochemistry parameters between the groups apart from a higher AST in AR- group (p=0.01).

Variable	AR+ (n=7)	AR- (n=17)	Ref. Int.	p-value
pH	6.0 (5.0-8.5)	7.0 (5.0-8.5)	5.0–9.0	n.s.
USG	1.020 (1.010-1.041)	1.027 (1.011-1.049)	1.001–1.060	n.s.
Protein (mg/dL)	25 (0-500)	150 (0-500)	NEG	n.s.
Bilirubin (mg/dL)	1 (0-1)	1 (0-6)	NEG	n.s.
Hemoprotein (ery./μL)	250 (150-250)	250 (0-350)	NEG	n.s.
WBC (/HPF)	5 (1-100)	7 (0-500)	0-3	n.s.
RBC (/HPF)	38 (10-100)	70 (0-250)	0-2	n.s.

Table S3: Quantitative urinalysis findings for AR+ and AR- PRAD tumors. There were no significant differences in urinalysis quantitative parameters between the groups.

Interpretation	AR+ (n=7)	AR- (n=17)	Ref. Int.	p-value
Alkaluria	2 (29%)	3 (18%)	pH > 7.5	n.s.
Aciduria	1 (14%)	1 (6%)	pH < 6.0	n.s.
Adequate USG	6 (86%)	16 (94%)	1.014 < USG <1.030	n.s.
Isosthenuria	1 (14%)	1 (6%)	USG 1.007–1.013	n.s.
Hyposthenuria	0 (0%)	0 (0%)	USG < 1.007	n.s.
Proteinuria	3 (43%)	11 (65%)	>75 mg/dL	n.s.
Bilirubinuria	5 (71%)	9 (53%)	>0 mg/dL	n.s.
Hematuria	7 (100%)	13 (76%)	>2 RBC/HPF	n.s.
Pyuria	4 (57%)	10 (59%)	>3 WBC/HPF	n.s.
Crystalluria	1 (14%)	9 (53%)	NONE	n.s.
Casts	2 (29%)	6 (35%)	NONE	n.s.
Epithelial cells	4 (57%)	9 (53%)	NONE	n.s.
Bacteriuria	2 (29%)	2 (12%)	NONE	n.s.

Table S4: Urinalysis interpretations and associations for AR+ and AR- PRAD tumors. There were no significant associations between urinalysis interpretation and AR status of PRAD tumors.

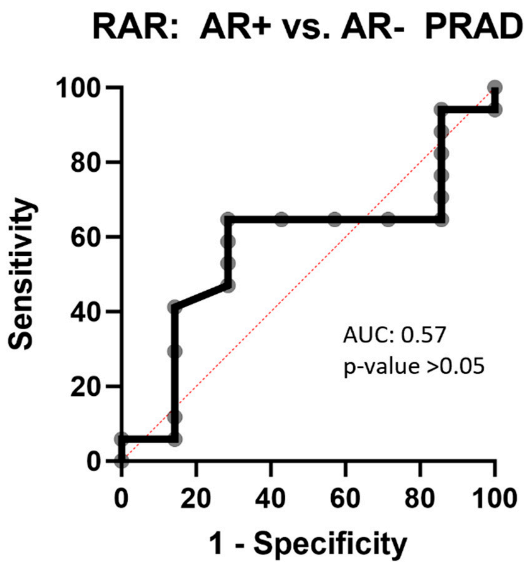

Figure S2: RAR is not significant in distinguishing AR+ from AR- PRAD.

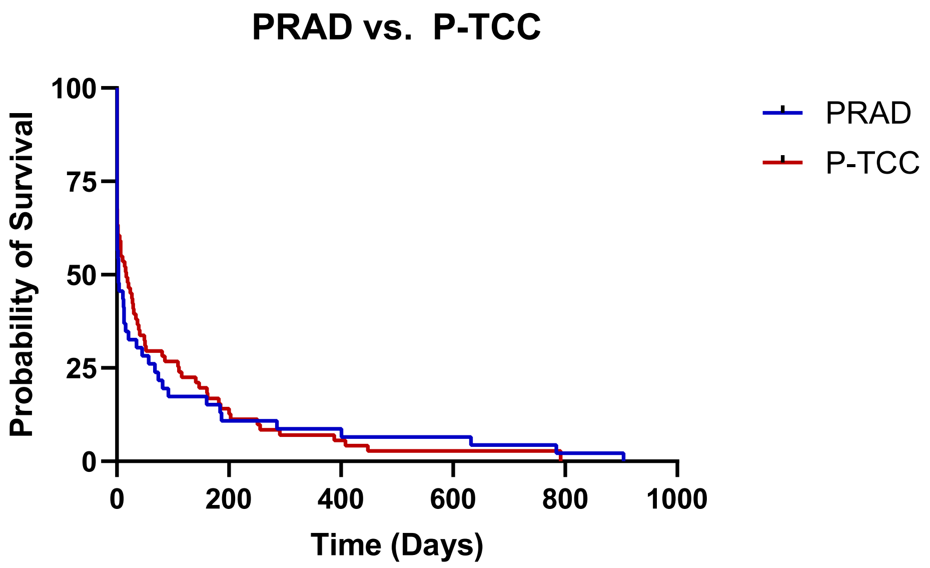

Figure S3: Survival times do not significantly differ between PRAD and P-TCC.

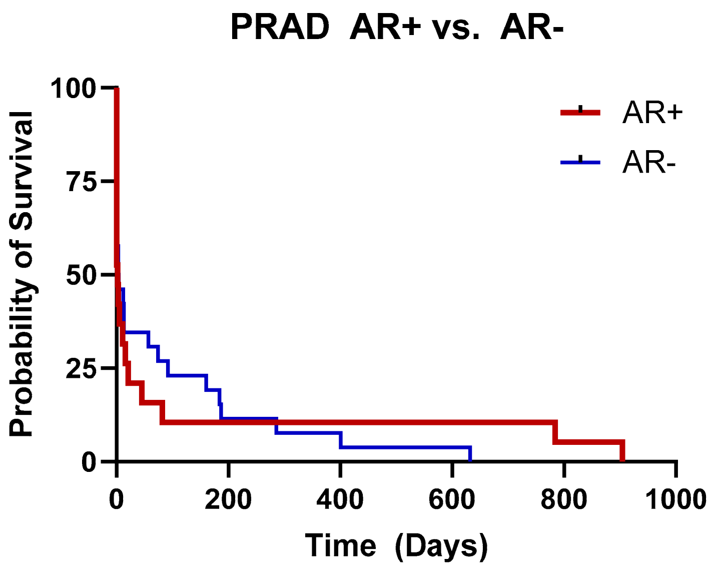

Figure S4: Survival times do not significantly differ between AR+ and AR- PRAD tumors.

Survival Time	PRAD (n=48)	P-TCC (n=67)
0 Days	18/48 (38%)	25/67 (37%)
1-30 Days	15/48 (31%)	15/67 (22%)
31-60 Days	3/48 (6%)	7/67 (10%)
61-90 Days	3/48 (6%)	2/67 (3%)
91-180 Days	3/48 (6%)	7/67 (10%)
181-365 Days	3/48 (6%)	7/67 (10%)
>1 Year	2/48 (4%)	3/67 (5%)
>2 Years	1/48 (2%)	1/67 (2%)
>3 Years	0/48 (0%)	0/67 (0%)

Table S5: Ranges of survival times in PRAD and P-TCC dogs. Many animals were euthanized the same day as diagnosis.

PCa Type	Variable	Hazard Ratio (95% CI)	p-value
PRAD	RAR (High, >4.11)	0.49 (0.06-2.95)	0.46
	RDW (High, >14)	0.714 (0.11-5.76)	0.73
	ALB (Low, < 3.4)	11.79 (1.99-102.93)	0.01
P-TCC	RAR (High, >4.11)	1.00 (Undetermined)	>0.99
	RDW (High, >14)	7.9×10^{-13} (Undetermined)	>0.99
	ALB (Low, < 3.4)	1.00 (Undetermined)	>0.99

Table S6: Multivariate survival analysis of 13 PRAD and 20 P-TCC dogs surviving >1 day.
